# Complementarity of Spike- and Rate-Based Dynamics of Neural Systems

**DOI:** 10.1371/journal.pcbi.1002560

**Published:** 2012-06-21

**Authors:** M. T. Wilson, P. A. Robinson, B. O'Neill, D. A. Steyn-Ross

**Affiliations:** 1 School of Engineering, University of Waikato, Hamilton, New Zealand; 2 School of Physics, University of Sydney, Sydney, New South Wales, Australia; 3 Brain Dynamics Center, Sydney Medical School – Western, University of Sydney, Westmead, New South Wales, Australia; Institut Science du Mouvement, CNRS, UMR6233, France

## Abstract

Relationships between spiking-neuron and rate-based approaches to the dynamics of neural assemblies are explored by analyzing a model system that can be treated by both methods, with the rate-based method further averaged over multiple neurons to give a neural-field approach. The system consists of a chain of neurons, each with simple spiking dynamics that has a known rate-based equivalent. The neurons are linked by propagating activity that is described in terms of a spatial interaction strength with temporal delays that reflect distances between neurons; feedback via a separate delay loop is also included because such loops also exist in real brains. These interactions are described using a spatiotemporal coupling function that can carry either spikes or rates to provide coupling between neurons. Numerical simulation of corresponding spike- and rate-based methods with these compatible couplings then allows direct comparison between the dynamics arising from these approaches. The rate-based dynamics can reproduce two different forms of oscillation that are present in the spike-based model: spiking rates of individual neurons and network-induced modulations of spiking rate that occur if network interactions are sufficiently strong. Depending on conditions either mode of oscillation can dominate the spike-based dynamics and in some situations, particularly when the ratio of the frequencies of these two modes is integer or half-integer, the two can both be present and interact with each other.

## Author Summary

We develop and demonstrate a model that allows us to examine how the predictions of spiking and rate-based models of neurons and their interactions are related. First, the behavior of a chain of neurons is explored by simulating each spiking neuron and spike-mediated interactions between neurons individually. Second, the same chain is studied using approximations based on the firing rate of the neurons. The predictions for these two approaches are closely compared and it is found that the simpler, rate-based approach captures the major system behaviors of the spike-based approach, namely spiking rates and modulations in those rates. Strong interactions between these modes take place when the frequency of one mode is an integer or half-integer multiple of the frequency of the other mode.

## Introduction

The brain is a multiscale system, whose dynamics spans from microscale structures, such as ion-channels and synapses, to emergent behavior, such as oscillations at the whole-brain scale. The problem then arises of how to simultaneously incorporate these diverse scales to make predictions about brain dynamics.

Neuronal dynamics has most often been studied by starting from single-neuron perspective via Hodgkin-Huxley equations [1] and their many variants for different neural types (e.g., [2], [3]), or via idealized models such as integrate-and-fire and binary neurons. Strong nonlinearities are responsible for spiking, with the spike cycle often described in terms of a nonlinear oscillator [4], [5]. Such approaches have been extremely successful in accounting for neural dynamics at the single- or few-neuron level.

Single-neuron approaches can also be applied to networks of many neurons by incorporating their synaptic interconnections. While very large networks can be simulated if sufficient computer power is available [3], [6], [7], the results of brute-force simulations can be difficult to interpret, especially when emergent network-level phenomena are involved. Moreover, common misconceptions that arise from the single-neuron viewpoint sometimes impede understanding of large-scale dynamics. For example, the starting-point picture of spiking being due to a nonlinear oscillator often leads to a focus on coupled-oscillator descriptions of neural interactions. If overemphasized, this can obscure the existence of (often linear, or near-linear) *collective* modes of oscillation in the network, which modulate spike *rates* at frequencies that are not related to the spike rate itself [8]–[10] — in general, both nonlinear-spiking and collective-oscillation phenomena exist. Some widespread errors in the literature that stem from this standpoint (when adopted naively) are: (i) that large-scale brain rhythms and electroencephalographic (EEG) oscillation frequencies must correspond to spike rates of specific neural “generators” or “pacemakers”, whereas they are quite different from spike rates in general, and (ii) that brain rhythms and EEG oscillations must be highly nonlinear because spikes are, whereas collective oscillations that modulate firing rates can actually be linear, or very nearly so [10]. Of course, collective oscillations can also have their own large-amplitude nonlinearities that survive averaging over spike generation, or arise through other effects [11]–[13].

An alternative starting point is to average over neural properties at the outset to obtain a neural field theory (NFT) [10], [14] in which the average dynamics of large numbers of neurons are modeled. In this case, instantaneous local firing rates are tracked, but individual neuronal spike dynamics are not. Such approaches are well suited to studying large-scale phenomena and bridging across scales and are much less computationally intensive than corresponding studies based on direct computation of single-neuron dynamics. However, as noted, they do not directly incorporate spiking dynamics of individual neurons.

Two aspects are of particular significance here. One is the internal dynamics of neurons. In this study, this is discussed in terms of a comparison between spike events described by *changes in membrane potential* (in the spike-based approach) and *spike rates* (in the rate-based approach). Communication between neurons is also critical. In the spike-based approach spikes travel between neurons that are coupled pairwise or via a field that carries spike profiles [14]; in the neural field theory communication is through propagation of fields that carry the spike rate only. In this work, we examine two limiting cases, one in which spiking neurons communicate via spikes, and one in which populations of neurons with rate-based internal dynamics communicate via rates — and make the dynamics as similar as possible in all other respects by having the same type of field carry either spike profiles or spike rates in the respective cases. In other words, one case involves spiking dynamics of neurons coupled by spikes carried by fields, and the second involves rate dynamics of continuous neural matter coupled by rates carried by fields; the fields obey the same propagation equations in both cases.

It is important to understand the relationships between the two limiting approaches, especially because they are complementary, not mutually exclusive. It is thus essential to understand when each is appropriate to be used, whether there are phenomena to which both can be applied, and which is the more convenient and tractable in given cases. Moreover, there can be situations where a fuller understanding requires an application of both approaches. This is analogous to situations arising in many other branches of science. For example, the properties of materials can be studied from a molecular viewpoint but, when dealing with large numbers of molecules, statistical approaches or continuum approximations are more convenient and appropriate starting points for obtaining understanding at the scales of most relevance — hydrodynamics is usually studied in terms of fluids, not molecules, for example. Likewise, statistical mechanics of particles passes over into thermodynamics for many applications as the number of particles becomes large, and there are intermediate regimes that can be addressed using either formalism, or variants such as nonequilibrium thermodynamics.

Some work toward understanding the complementarity of spiking and mean-field approaches has been done, in part by developing hybrid models that preserve aspects of both single-neuron and mean-field approaches. For example, Robinson et al. [15] and Wu et al. [16] showed how to write the spike rate of Wilson neurons [2] in terms of the spike rate itself (rather than instantaneous cellular voltages), thereby eliminating the need to track individual spikes if rate is all that is desired. This work put the Wilson model of spiking and bursting neurons [2] in a form suitable for incorporation into NFT and allowed top-down systems-level influences on single neurons to be analyzed tractably. The predictions of this NFT were subsequently investigated for a model system incorporating a simple delayed feedback loop whose resonances could interact with natural neural spiking and bursting frequencies [16]. Robinson and Kim have very recently developed a series of hybrid methods of treating neural interactions that combine various aspects of spike- and rate-based neural dynamics and of the discrete vs. mean-field features of spatial coupling [14]. Bressloff and Coombes have shown how fluctuations in firing rates consistent with a neural field model can be produced by a network of integrate-and-fire neurons particularly when slow interactions are present [17].

There are other approaches to neuronal modeling which we mention for completeness. The population density approach (e.g. [18]) moves beyond a model based on mean firing rates alone by considering the changes in the distribution of neuron properties. In the population density approach, individual neurons or groups of neurons are not modeled explicitly, rather the change in the probability density function of the state of the neurons is modeled. This approach can be many times faster than a direct Monte Carlo simulation of neurons or groups of neurons. One can also focus on the correlations and higher-order moments of a distribution. This is of significance since correlations in activity may form a significant part of the mechanism through which neuronal signals carry information. Recently, Touboul and Ermentrout [19] have studied the correlation approaches of Bressloff [20] and Buice et al. [21] and shown them to be equivalent when applied to a system of infinite size. This allows large networks of neurons to be analyzed. Significantly, by considering correlations rather that just mean firing rates, dynamical behaviors can appear that cannot be accounted for with a lowest order mean field approach alone.

However, the specific aim of this paper is to investigate and elucidate the complementarity between spike- and rate-based approaches to neural dynamics by use of an overarching approach that can accommodate both pictures in the analysis of a test system that is suited to exposing the key phenomena. Although other approaches may also be informative, we focus on the complementarity between spike- and rate-based simulations in the current work. We begin by reviewing the theoretical background and developing our model. We then present the numerical methods, and give the results of our analyses. We analyze, compare and contrast the dynamics of the spike-based and rate-based approaches. Finally, we interpret the results and discuss their applicability and significance. For simplicity, homogeneous models are used; however, the methods discussed are generalizable to inhomogeneous situations.

## Models

### Neural Field Theory

In this section we briefly outline the NFT equations required, specializing the treatment to a specific, idealized test system. The model we use is that of a single cortical population, driven by an external drive and incorporating direct interactions between neurons and indirect ones via a delayed feedback loop.

**Figure 1:**
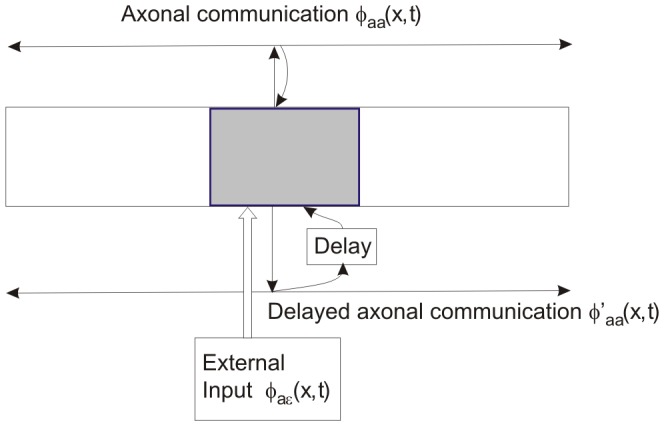
Detailed schematic of the model system. The gray box denotes a neuron from population 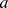 at a position 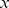. This neuron communicates with others in the population through axons; the upper axon in the figure describes 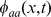; the lower describes 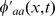. The neuron receives both immediate (top) and loop (bottom) feedback from the axons. It is also driven by input externally by 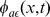.

We consider the system of interconnected neurons which includes synaptic input to a set of neurons (labeled by a suffix 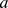) from an external set of neurons (suffix 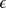). The former set consists of a one-dimensional chain of neurons with periodic boundary conditions, and has a feedback both directly 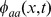 and via a loop 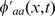, where 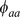 and 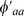 are the rates of incoming spikes at each synapse (i.e. have dimensions of inverse time), and 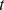 is time. The loop features a feedback delay time 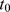, and the feedback is assumed to be topographically organized (i.e., each point in space feeds back most strongly to itself). This idealized system is sufficiently general to study complementarity between rate- and spike-based treatments; it is also easily generalized to include more types of neurons and higher dimensionality [22], [23]. Biologically, such topographical feedback is found in the thalamocortical loop. Excitatory neurons in the cortex drive the coupled thalamocortical and thalamic reticular neurons of the thalamus; in turn the thalamocortical neurons project back to the cortex in a manner such that a signal returns very close to where it originated [24]. The spiking model is summarized in graphical form in Fig. 1.

When applied to real brain tissue, NFT averages neural properties over linear scales of a few tenths of a millimeter, sufficient to embrace many neurons [10], [22]. The soma potential 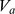, measured *relative to its resting potential*, responds to spikes via synaptic dynamics, dendritic signal dispersion, and soma capacitance. The resulting response to synaptic input approximately obeys [10], [22], [25], [26]

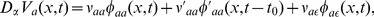
(1)where
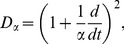
(2)

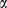
 is the mean response rate of 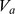 to synaptic input, 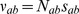 is the mean connectivity strength to neurons of type 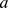 from those of type 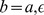, 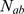 is the corresponding mean number of synaptic connections, and 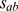 (with dimensions voltage times time) is the mean strength of these connections, defined to be the time integral of the postsynaptic potential change due to a spike afferent on a neuron 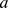 from one of type 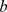.

Action potentials are produced at the axonal hillock when the soma potential exceeds a threshold [10], [22], [25]. When averaged over a local population of neurons, a good approximation to the firing rate is
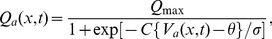
(3)where 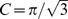, 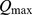 is the maximum firing rate, and 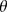 and 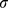 are the population mean and standard deviation of the threshold [10], [22], and 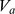 is the mean soma potential averaged over a local population of neurons. We discuss the origins of this relationship below.

Prior work has shown that the mean fields of axonal signals, 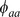, 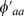, and 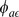, propagate approximately as if governed by damped wave equations [22], [27], [28], one form of which is
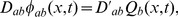
(4)where
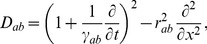
(5)

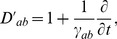
(6)where 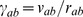, with 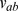 the axonal velocity, and 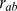 the characteristic axonal range [22], [27].

Equations (4)–(6) incorporate spatiotemporal coupling between neurons. This is more easily seen through the corresponding Green-function (i.e., propagator) formulation [22], [26]:
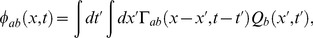
(7)

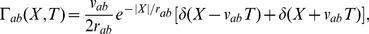
(8)for 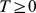, with the Green function satisfying 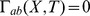 for 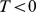 to ensure causality, and where translation invariance of the system has been assumed. We choose the form (8), which follows from (5) and (6), to preserve the timing and shape of narrow pulses in one dimension, since we want to compare spike-based coupling with field-theoretic coupling in the present work.

The spatial coupling vs. 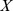 is found by integrating (8) over 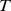, [22] which gives
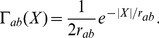
(9)Integrating (9) over 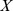 yields a normalization of unity, which reflects the fact that each pulse that enters an axon ultimately reaches its end. Equations (4)–(6) thus represent signals that propagate along axons at a uniform velocity 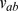, but where the number of axons reaching a distance 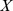 decays exponentially as a function of 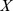, with characteristic range 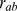. This is a reasonable first approximation to the coupling of cortical neural populations by axons in one dimension. If we were to replace (6) by 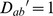, we would recover the form introduced in by Robinson et al. [22], which yields broader temporal pulses in response to a delta input. The latter form is actually more realistic in general, especially in two dimensions, since axons are neither identical in velocity nor exactly straight, thereby making delta-function propagation of a mean pulse field very much an idealization. Here we retain (6)–(8) to obtain (9), which is commonly assumed in spike-based analyses. Moreover, this form provides a more stringent test of complementarity with rate-based analyses because it involves no temporal smoothing of the propagated signal, which would tend to make the two cases more similar.

Delayed integrodifferential equations such as these have been well studied, both in general and in the context of neuronal modeling [29]–[31]. The presence of delayed feedback leads to Hopf bifurcations and other dynamic phenomena such as traveling waves [32]. We expect to see such features in the models discussed here.

### Spike Based Theory and Link with Neural Field Methods

We now briefly review the Hindmarsh-Rose fast-spiking neuron model [2], [33], [34], and how it can be put in a form compatible and comparable with NFT. Conductance-based equations for the rate of change in membrane potential 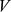 in a *single* fast-spiking neuron, appropriate to the mammalian neocortex, can be written [2], [14]–[16], [25], [33], [34]

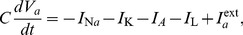
(10)

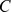
 is the capacitance per unit area, 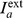 is an externally imposed input current per unit area (e.g., due to synaptic input from other neurons), 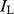 is a leakage current per unit area, and 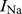 and 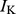 are the 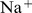 and 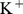 currents per unit area, respectively, and 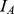 is a transient potassium current that enables these neurons to fire at very low spike rates when 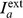 is small. Note that use of the script font 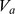 indicates a voltage measured relative to the extracellular fluid (i.e., a membrane potential) rather than a measurement taken relative to the resting state — there is a constant offset between 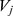 and 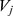 equal to the resting potential 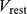; i.e., 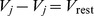. Each of the currents is assumed to obey Ohm's law, with
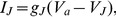
(11)where 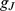 is the conductivity per unit area and 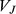 is the equilibrium potential of the ion 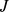.

Numerous authors have investigated Eqs (10) and (11) for fast-spiking neurons, the main population in the mammalian neocortex, and have found simplified expressions for their dynamics, which can be closely approximated by just two equations that gave an adequate description of spiking dynamics [2], [33]–[36]. There is one equation for the membrane voltage and one for a dimensionless recovery variable 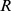 that describes the coupled opening of 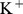 channels and corresponding closure of 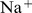 channels [2], [34]. The equations are
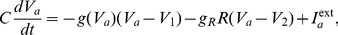
(12)

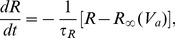
(13)where
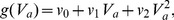
(14)

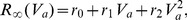
(15)with 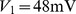 being the 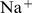 reversal potential, 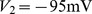 the 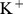 reversal potential, 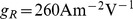, 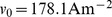, 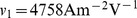, 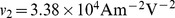, 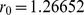, 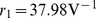, 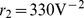, 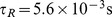, and 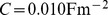 for fast-spiking neurons.

The dynamics of (12) and (13) have been discussed in detail elsewhere (e.g., [2]), so we summarize very briefly here. At low 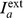 they have three steady-state solutions: at 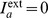 these are a stable node with 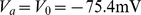 and 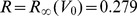, an unstable saddle point at somewhat higher 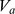, and an unstable spiral point at still higher 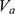. The first of these represents the resting (non-firing) state. As 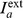 increases, the two lower fixed points approach one another, then generate a saddle-node bifurcation when they coalesce at the critical current 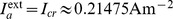 with 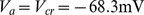 and 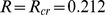. This gives rise to a limit cycle that encircles the resulting spiral point. Each orbit of the limit cycle corresponds to the generation of one spike; hence the picture of spike generation being due to a nonlinear oscillator.

The frequency of the limit cycle (i.e., the firing rate) satisfies [5], [15], [37]

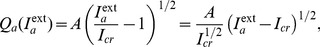
(16)for 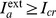 and 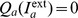 for 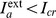, which corresponds to a continuous increase from zero firing rate as 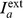 increases beyond 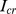. Simulations show 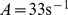 in Eq. (16) [2], [15], [34].

We next show that we can couple individual model spiking neurons together in a way that can be compared directly with NFT of the same system. In NFT, the mean membrane potential of a population of cells is driven by the incoming axonal pulse rate. However, in the spike theory, membrane potential is driven explicitly by current entering the cell body from the dendritic tree. Standard cable equations imply that this current is proportional to minus the spatial derivative of the voltage at the soma boundary. Hence, the functional form of the driving current to a cell 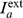 induced by a delta function spike at a synapse has the same temporal dependence as 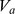, apart from a dimensional constant of proportionality [15], [25]. Thus, it obeys
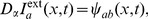
(17)where 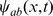 is the time course of the part of the afferent signal that is above the channel opening threshold and 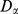 is defined in Eq. (2). This can be approximated as
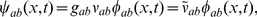
(18)in NFT notation, where the quantities 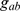 (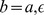) have units of conductance per unit area and the connection strengths 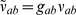 have been introduced. These incorporate the membrane conductance per unit area and will be used in the model to control the relative strengths of the direct and loop feedback, and external drive. Henceforth, we discuss the model in terms of connection strengths 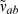 rather than the 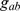. This formulation allows communication between neurons via the intermediate fields 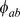 (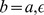), which can propagate spike profiles, not just average rates, provided we now replace (4) by
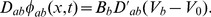
(19)where 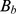 is a constant which we determine shortly. That (19) reproduces (4) can be seen by averaging (19) over timescales much longer than a spike width. Explicitly, averaging Eq. (19) over the inter-spike interval 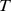 (which varies as a function of space and time) gives
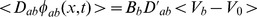
(20)where the angle brackets denote the average over 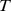. Since 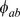 changes over time-scales much longer than a spike, we can write 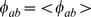, leaving
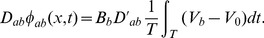
(21)If
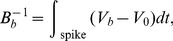
(22)then it is clear that
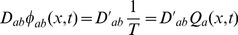
(23)where 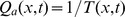 is the spike rate. Here we have assumed that the integral over 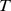 is only significant within the vicinity of the spike, i.e. 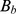 can be caluclated from Eq. (22) by considering a stereotypical spike profile. We assume (22) henceforth.

In dealing with rates in populations of neurons the idealized square root form (16) of the response curve discussed earlier must be convolved with a distribution (e.g., a Gaussian) of some width 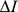 that encapsulates fluctuations in the properties of the neurons and their input: e.g., variations in number and strength of synaptic connections, and in the various channel conductances, especially from neuron to neuron. Such convolutions smear (16) over a width 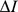 [15]. A further source of broadening is fluctuation in arrival rate of spikes and associated changes in membrane voltage [38]. A good approximation that also captures saturation effects is the sigmoidal function
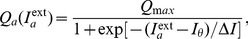
(24)which is equivalent to the rate-voltage relationship (3) via
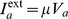
(25)where 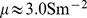 [15] is a conductance per unit area and 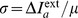.

### Numerical Implementation of Spike Based Equations

We now in a position to write explicitly a set of coupled differential equations for our 1D chain of identical neurons, in a form that is consistent with NFT in the relevant limit. For each neuron at a point 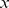 in space, we use the Wilson neuron model to describe its membrane potential 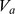 and recovery variable 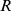. We emphasize here that the spikes are carried through a field rather than through pairwise interactions, which corresponds to the neuron-in-cell approach recently introduced by Robinson and Kim [14].

The input 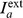 to the neuron comes from both synaptic input from other neurons (through a current term 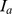, which is explicitly modeled below), and the input from the external drive term, labeled 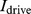. The set of coupled differential equations is now obtained from (12) and (13) for the neural dynamics, (17) and (18) for the synaptic dynamics, and (19) for the propagation of fields along axons. To model a level of random external inputs, a white noise current density term 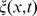 is added to the neural dynamics on a grid, where 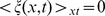 and 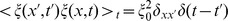 with 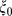 a constant. The resulting equations are
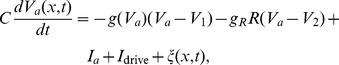
(26)

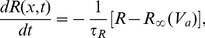
(27)

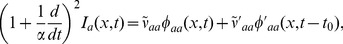
(28)

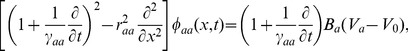
(29)

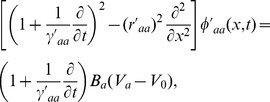
(30)where 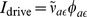 is a constant external drive. The variables 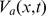, 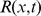, 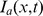, 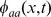, and 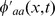 describe the state of the system, with 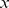 distinguishing the locations of the neurons. Values of constants used in this paper are mostly taken from previous work [2], [23], [39] or, in the cases of 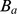, 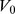, 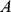 and 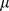, numerical analysis of the Wilson model neuron [2], and are listed in Table 1. The level of noise, through the parameter 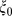, is chosen so that fluctuations are small and linear approximations are valid when used. Here we have separated the direct and loop feedbacks (29) and (30), respectively, to enable the use of 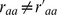, and 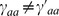 in general.

**Table 1:** Table of constants.

Symbol	Value	Unit
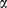	60	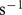
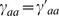	80	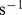
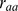	0.04	m
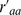	0.01	m
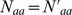	4120	–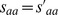	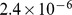	V s
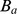	8696	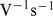
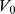	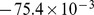	V
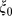	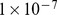	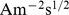
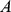	33	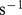
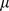	3.0	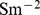

Values of the constants used in the spike based equation model of Eqs (26)–(30). The constants used in the equations for 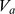 and 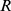 of the Wilson model [2] are given by Eqs (12)–(15); the parameters 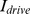, 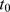, 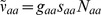, and 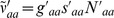 are varied in the simulations. The constants 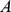 and 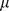 are used only in the mean field approximation of these spike based equations. The constants 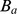 and 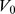 come from simulation of the Wilson neuron model [2].

Before we discuss the numerical implementation of the equations we emphasize that we have not proved that there are well-behaved solutions to these. However, wave equations are well understood physically and numerically; e.g. [22], [27], [28], [40]. Moreover, numerical simulations as discussed below produce results that do not diverge with time. In numerical implementation of the model, the Eqs (23)–(27) are discretized on a 1D spatial grid. When spatially discretizing, several issues must be considered: (i) we must ask whether the numbers of neurons and system size are sufficiently large to ensure results adequately represent real brain dynamics and are not numerical artifacts. The suitability of the number of neurons can be estimated by asking the question of how many input spikes are needed to generate an output spike. In the human brain this is large, with each neuron receiving input of order 10 spikes per second at each of thousands of synapses. Overall, if the effective soma integration time leading to a spike is 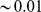 s, several hundred presynaptic input spikes contribute to each postsynaptic spike [41]. In our simulations with 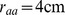, each neuron is locally coupled to neighboring neurons in approximately 8 cm of tissue. By placing neurons approximately 0.25 cm apart, this gives us 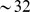 locally coupled neurons. Typically in the simulations neurons fire at a rate of 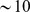 spikes per second, giving 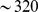 spikes arriving at each neuron per second, implying that each spike is generated as a result of a neuron receiving 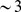 input spikes during the relevant integration time. This is much lower than in the cortex; however, computational demands, which scale linearly with the number of neurons, necessitate the use of relatively few neurons. However, we have also carried out some larger runs with considerably more sampled spikes, to begin to explore the effects of relaxing this limitation. No qualitative difference is observed, suggesting that our levels of temporal and spatial discretization are sufficient. (ii) We also anticipate that a system that is too small would introduce artifacts: e.g., with periodic boundary conditions if the system is too small, long-wavelength modes of activity are not captured. Moreover, a model that is of order 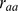 or smaller in size would be affected by wrap-around of connections through the periodic boundary conditions. However, biologically, it should be remembered that the cortex is not of infinite size; the ratio of 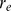 to cortical radius is approximately 0.6
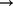
1. A system size of 20 cm is used for most runs; this is adequate in terms of removing numerical artifacts and computer resources and does not represent an implausible size biologically. Some simulations have been carried out with a larger system size and results are not significantly different.

Initially, the variables 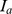, 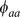 and 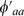 are assigned the value zero for all spatial points. The membrane potential 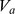 and recovery variable 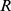 are assigned the values they would have at equilibrium when no external current is applied, namely 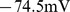 and 0.279 respectively. The equations are integrated forward in time with a second-order stochastic predictor-corrector method [40]. In order to generate initial activity a high driving current is applied for the first second of simulation and then removed. The Courant condition requires that the time step 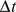 must be smaller than the grid spacing divided by the velocity of a pulse to ensure numerical stability [42]. The typical step size of 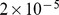 s is comfortably within this limit.

### Neural Field Approach

We also treat the system of Fig. 1 using the complementary neural field approach of coupling neurons using rate of firing, rather than individual spikes. These rates are propagated using the same Green functions (and same wave equations) as for spikes in the spike-based approach, but individual spikes are not tracked.

In the NFT approach, each grid point is taken to represent the average dynamics of a local population of neurons. To do this we replace the equations for the membrane potential (26) and recovery variable (27) with a single equation that relates the firing rate 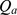 to the input current, via the square-root function (16), whose parameters were calibrated to reproduce the dynamics of fast-spiking neurons in previous work [14]–[16]. This *rate* is used to provide input to the wave equation, rather than using the potential term explicitly. A small amount of white noise 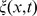 is added to the current, where 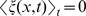, 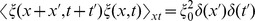, where 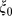 is a constant. The noise provides a small perturbation to the system to allow it to quickly explore phase space and ensure that no two simulations are identical. Therefore we obtain the following nonlinear set of four coupled equations for the variables 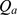, 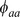, 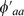 and 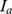.
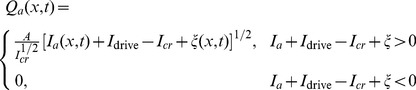
(31)

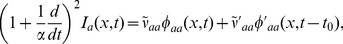
(32)

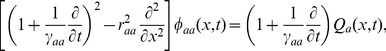
(33)

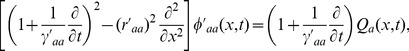
(34)Numerically, this set of neural field equations can be integrated forward in time using the same approach as for the spike-based case. In this case, the time step can be made larger than for the spike based model, although subject to the Courant condition for numerical stability, because the spike profiles are not modeled explicitly. This is a major advantage of field-based approaches over spike-based approaches.

Equation (31) is appropriate rather than Eq. (24) since for simplicity we will consider homogeneous parameters. Equation (31) allows us to compare explicitly the firing rates predicted by the neural-field approach to those of the spike-based approach. If inhomogeneous parameters were used, Eq. (24), with values of 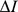, 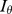 and 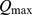 specific to the parameter distribution used, would be appropriate.

Equations (31)–(34) can be used to compute the firing rate at various points in space and time; i.e., the mean firing rate of all neurons in the vicinity of each grid point vs. time. Hence, for this to give a good representation of average dynamics, each grid point should correspond to multiple neurons. In the present case, this means the separation of grid points in the neural field model must be much larger than the length scale between neurons, which is satisfied in the present work. Therefore, in carrying out detailed comparisons between the spiking model and the field model, the results of the spiking model need to be coarse-grained (i.e., averaged over the appropriate length scale). We again emphasize that in this work we have carried out simulations at various length scales and neuron densities, and results are qualitatively unaltered by changing the scale (i.e. our discretization is fine enough for the purposes of this work).

### Linear Spectrum of NFT

It is found that the system (31)–(34) has at least one spatially uniform equilibrium state, which is obtained by setting all the temporal and spatial derivatives to zero. In general there may be one or three solutions (plus a special case of two solutions); however if 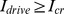 as in this work, there is only a single solution. Equations (33) and (34) can then be solved to obtain equilibrium values 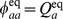, 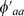 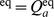, and so via Eq. (32):
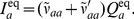
(35)Equation (31) gives
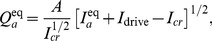
(36)for 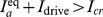, and 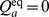 otherwise. Squaring Eq. (36) and substituting Eq. (35) for 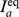 gives a quadratic equation for 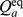 that is easily solved for the positive firing rate solution.

By writing the deviations from their equilibrium values of 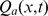, 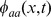, 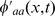, 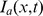, and the noise input 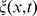 in terms of their Fourier components in both space and time, we can establish the power spectrum of fluctuations in both temporal frequency 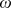 and spatial frequency 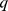. To calculate these quantities, we linearize Eqs (31)–(34) in small deviations from equilibrium, and write the Fourier form
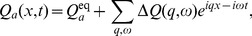
(37)with similar expressions for 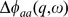, 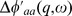, and 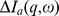. We also note that the noise has an equilibrium value of zero and omit the 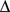 from 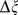 henceforth. This gives us the linearized equations
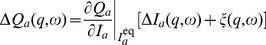
(38)

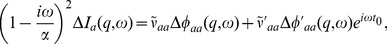
(39)

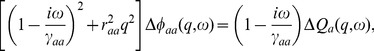
(40)

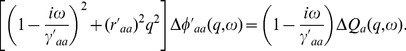
(41)These equations can be solved for 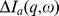 (or any other of the variables) in terms of the noise input 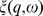 to give us 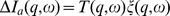, where the transfer function 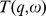 is given by
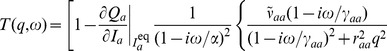


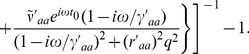
(42)


In general, Eq. (42) is difficult to analyze further analytically, especially because of the term 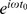. However, a useful limiting case can be seen when the system has only loop feedback whose time delay is much longer than the timing of the synaptic current pulses and wave events; i.e., 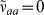, 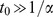 and 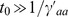. In this case we can make the approximations 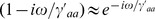 and 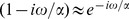 to give us the transfer function at 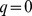:
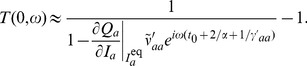
(43)The response (43) will have a resonance when the phase of the complex exponential is a multiple of 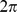, which gives resonances at angular frequencies of 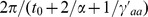 and its harmonics [16]. When both direct and loop feedback are present, these resonances modulate the combined spectrum to produce peaks, as found originally by Robinson et al. [43], [44].

### Synthesizing a Spike Train from the Neural Field Calculation

It might appear that use of a neural field model, where only spike rates are calculated, might remove all information about individual spike times; however, this is not the case [14]. Neural field theory yields instantaneous spike rates 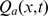 as functions of position and time, so the integral of the local rate over some time period 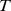 is the expected number of spikes 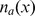 that occur at that location in this time period; i.e.,
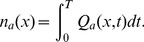
(44)Moreover, when the integral increments by one, we know that there must be exactly one spike during this interval, so
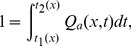
(45)defines the expected time 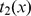 at which the next spike occurs, given that one occurred previously at 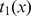. To construct a membrane potential time series from the spike timings 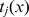, where 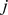 is an integer, one can write the potential 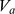 as a function of the noninteger part of the integral (44). Therefore,
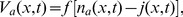
(46)where 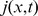 is the integer part of 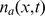 and 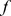 is a function that describes the spike profile. If required, this profile can be quickly computed from a look-up table [14]. Since this approach requires only the tracking of 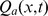 rather than spike profiles, it is computationally less intense than a spike-based approach, since larger time steps can be used. Implementing this approach requires initial phases to be specified for the neurons, or for enough time to pass that the system loses memory of its initial conditions.

Before presenting the results, we emphasize that in all cases *fields* are used to describe propagation of signals between neurons, either carrying spikes from neuron to neuron through Eqs. (29) and (30) or conveying rates fields between spatial locations through Eqs. (33) and (34).

## Results

Simulation of the spike-based equations (26)–(30) generates output of each of the state variables as a function of position and time. Particularly useful is the membrane potential 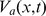 from which the times of firing of the neurons can be readily extracted. A plot of membrane potential vs. space and time gives an immediate representation of the system dynamics (e.g., synchronous firing, bursting, traveling waves of activity). In the NFT case, simulation of Eqs. (31)–(34) generates output for the state variables; the time series of the membrane potential 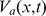 can then be reconstructed by the method described above.

Also useful is a Fourier space representation of the results, which enables robust identification of wave modes and, in particular, firing rates. One can in principle apply a Fourier transform in space and time to any one of the five state variables 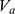, 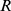, 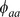, 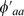 and 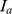 (for the spike-based case) or the four state variables 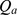, 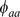, 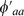 and 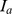 and the reconstructed potential 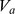 (for the NFT case). In this work we concentrate on the variables 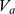 and 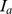. The former is most directly related to an experimentally measureable quantity, namely the membrane potential. The disadvantage of using 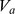 is that the highly nonlinear spike features lead to high frequencies in the spectrum that can mask the subtleties of subthreshold fluctuations. The latter is chosen since it is temporally the smoothest of the state variables and so its Fourier transform contains fewer features due to the nonlinearities and thus is most suitable for comparison with a linearized calculation.

The utility of comparing rate- and spike-based approaches via analysis of 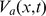 or 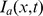 depends on the primary mode of behavior of the system. Where the spike-based model shows a spike-dominated behavior (e.g. spiking at a constant frequency) a Fourier analysis based upon 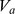 provides a meaningful comparison with the predictions from the NFT; where a rate-based oscillation dominates (e.g. spike rate fluctuates or depends upon time delay) a more appropriate comparison would be with the NFT predictions for 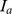.

Typically, simulations are run for a total time of 20 seconds. For the first second, a high external drive current is used, as this is sometimes required to initiate spiking in the system; after this time the drive current is removed. Typically, the first four seconds of each time series are discarded to exclude initial transients, the remaining time is split into short periods (typically 4 seconds). Each period is windowed by applying a Hamming window, then the spectrum of 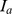 or 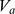 is calculated, as appropriate. The spectra are averaged over all the windows to produce a final power spectrum 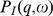 or 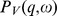. For the case of 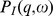,this can be compared with the power spectra predicted by the mean field result Eq. (42). In order to show the effect of individual model parameters on the results, we also show plots of the breathing-mode power spectrum [i.e., 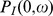 or 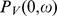] for various values of each such parameter of interest. A further analysis is the evaluation of the spatial correlation function 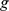, which is given by the inverse Fourier transform of 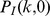 or 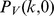 for the cases of current density and voltage, respectively.

Before exploring the parameter dependences of the model in detail, we first show a typical case, by way of illustration. In later subsections, the results of the model are illustrated with a variety of different cases. In particular, we compare the predictions of the spike-based analysis with the neural field approach to highlight similiarities and differences in behavior. We illustrate the change in behavior of the model system as a function of the key parameters (time delay 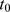, external drive current 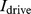, and direct and loop connection strengths 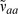 and 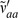) by keeping all but one parameter constant, and varying the others. We also present a comparison of the spiking events from the spike based model and a reconstruction from the neural field model.

### Illustrative Dynamics and Spectra

**Figure 2:**
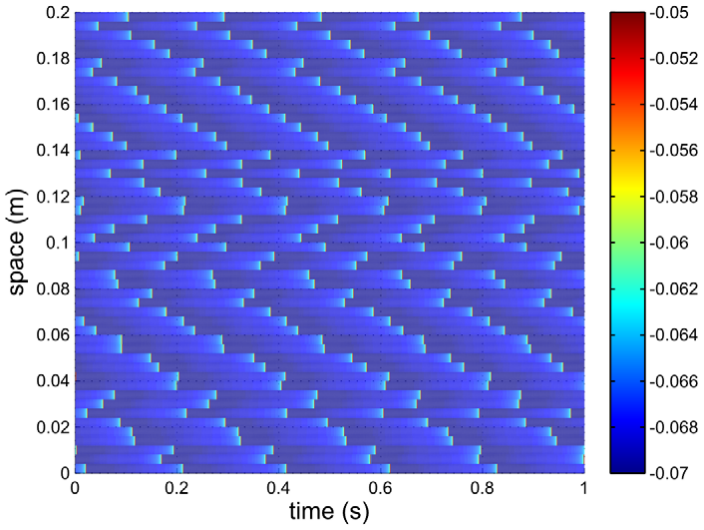
Typical neural firing pattern in voltage. Here 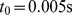, 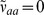, 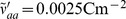, and 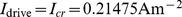. Colors denote membrane potential in V.

To start, we demonstrate typical behavior of the spike-based state variables 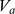, 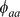, 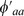 and 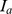. For this illustration we use a small positive loop feedback; i.e., 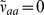, 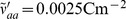. The external drive current 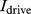 is chosen to be equal to the critical current 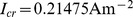. This external current would put an individual neuron at the point of spiking, so that the positive feedback between neurons ensures that they obtain a modest spike rate; this allows us to explore the interaction between spike-based and collective oscillations. Biologically, it is reasonable that a neural system can organize to be near a critical point [45], [46]. A very short time delay is used, 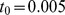 s so that delays between the direct and delayed feedbacks are negligible compared to the timescales of the dominant neural activity in this case (i.e., the interspike interval). Fig. 2 shows the membrane potential 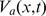 of each neuron over a typical 1 second period. The spike events are clearly shown, indicating a spike rate of about 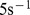. There is clearly evidence of spatial structure in the firing pattern, which we elucidate through the 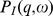 spectrum below.

**Figure 3:**
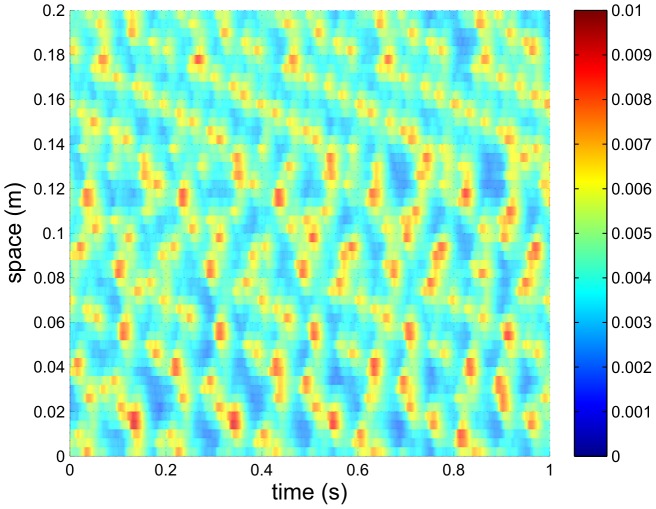
Typical neural firing pattern, showing the current 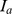 into the neurons for the same case as Fig. 2. Here 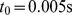, 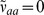, 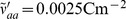 and 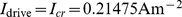. Colors denote current density in 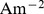.

The current 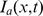 is plotted in Fig. 3. It is much more smoothly varying than the spiking voltage. However, firing events can still be discerned via their associated rapid increases in 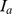 versus time, meaning that a firing rate, as opposed to modulation in rate, will be the most obvious feature on any spectrum.

**Figure 4:**
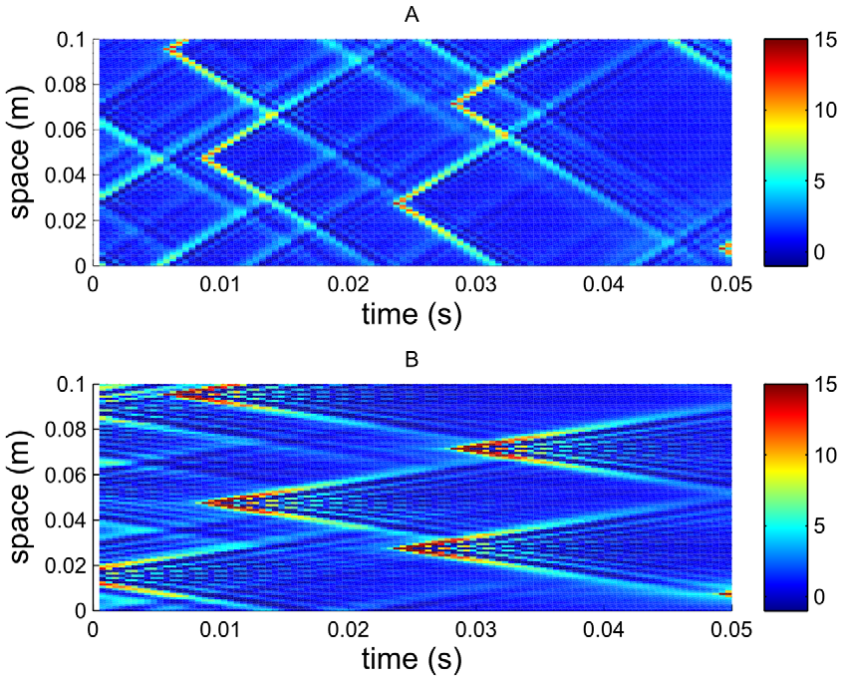
Typical axonal pulse field patterns, showing (A) 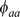 and (B) 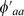 vs. time for the same case as Fig. 2. Here 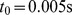, 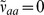, 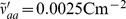, and 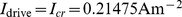. Colors denote rates in 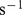.

The variables 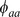 and 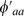 are shown in Fig. 4. In order to show the spatio-temporal structure of these fields, only a small part of the spatio-temporal domain is shown here. The scales are different for Figs. 3 and 4. These variables denote the propagation of signals between neurons. The plots show signals emanating from each firing event as ‘
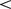
’-shaped features. The apex corresponds to the firing event, while the two arms of the ‘
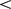
’ show the propagation of the signal forward in time at constant speed in both spatial directions. The gradient of the arms of the ‘
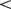
’ for the direct feedback term 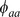 is 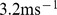, corresponding to the signal speed given by 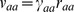; likewise, the gradient of the features for the loop feedback 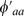 is 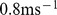, which equals 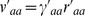. It is also clear from the length of the arms that the direct feedback events in Fig. 4(a) have a longer spatial range than the loop-mediated ones in Fig. 4(b), in accord with 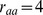 cm and 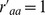 cm here.

**Figure 5:**
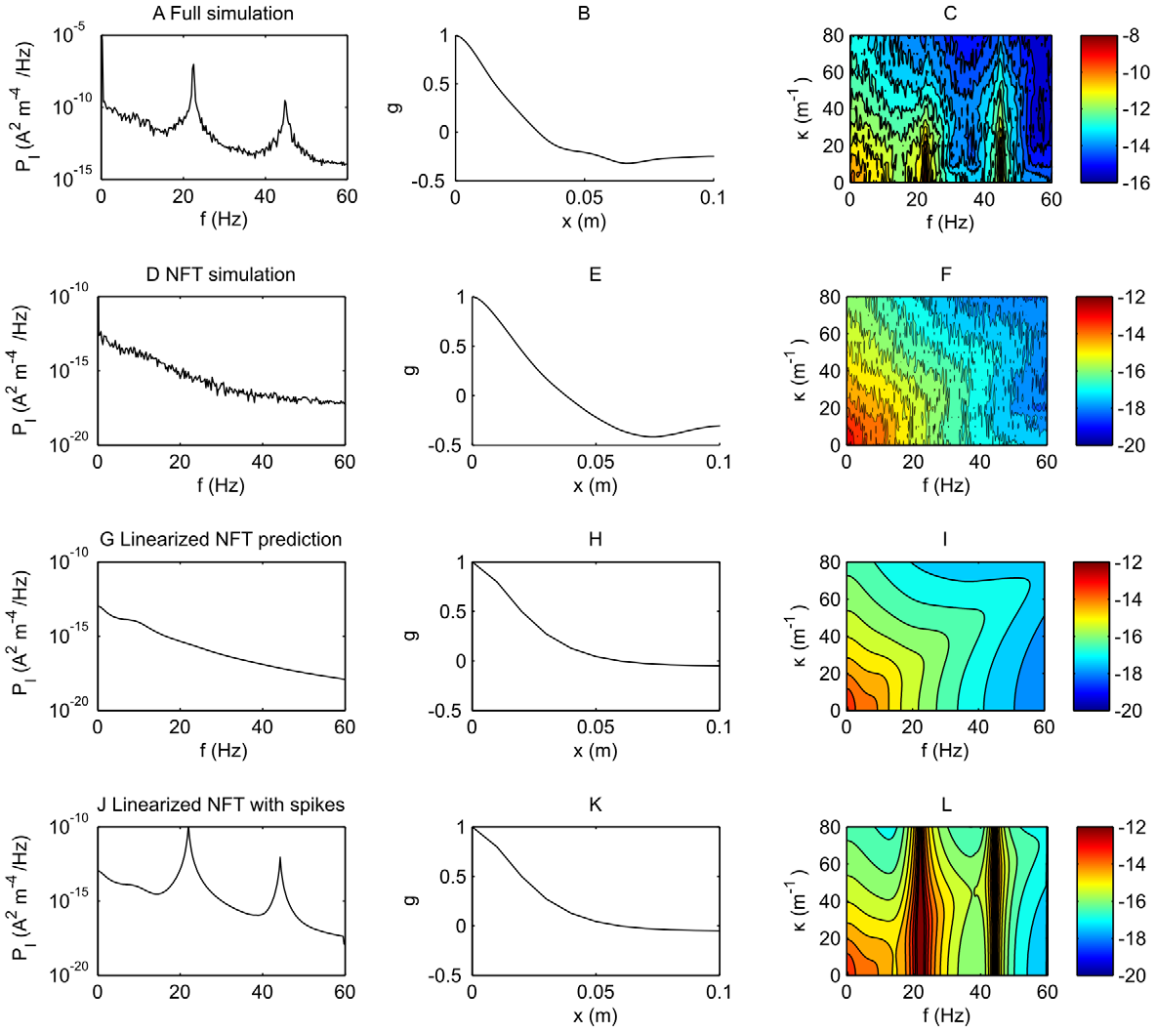
Comparison of the power spectra for current density for the spike based model and neural field predictions for a fast firing case. Here, 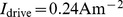, 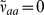, 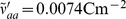, with 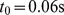. A. Power spectrum of the spike-based simulation at zero spatial frequency, 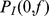. B. Spatial correlation function for the spike-based simulation. C. The spatio-temporal spectrum 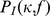 for the spike-based model on a logarithmic (base 10) scale. One contour represents half an order of magnitude change in power. D. Power spectrum of the NFT simulation at zero spatial frequency, 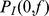. E. Spatial correlation function for the NFT simulation. F. The spatio-temporal spectrum 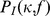 for the NFT simulation on a logarithmic (base 10) scale. G. Theoretical power spectrum at zero spatial frequency 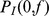 calculated from the transfer function Eq. (42) for the NFT at zero spatial frequency, 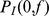. H. Theoretical spatial correlation function for the NFT calculated from the transfer function. I. The theoretical spatio-temporal spectrum 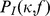 on a logarithmic (base 10) scale, from the transfer function. J. The theoretical power spectrum 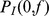 with spike features attached. K. The theoretical spatial correlation function. L. The theoretical spatio-temporal spectrum 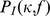 with spike features added.

**Figure 6:**
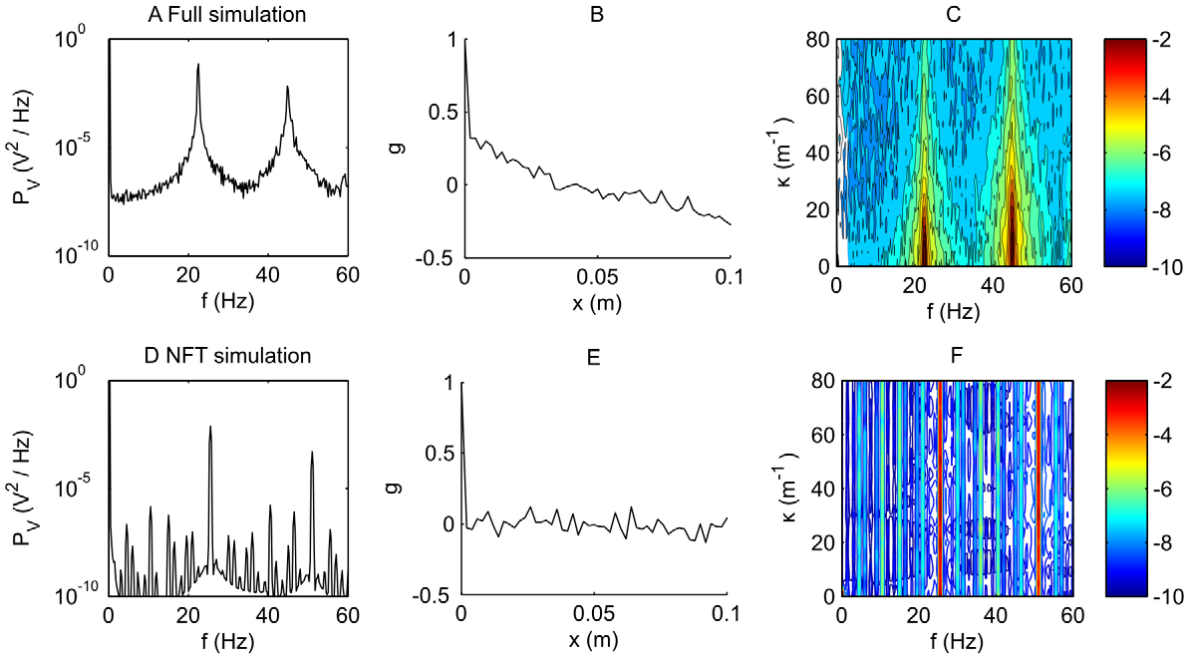
Comparison of the power spectra for soma voltage for the spike based model and neural field predictions for a fast firing case. Here, 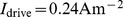, 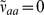, 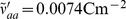, with 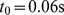. A. Power spectrum of the spike-based simulation at zero spatial frequency, 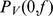. B. Spatial correlation function for the spike-based simulation. C. The spatio-temporal spectrum 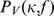 for the spike-based model on a logarithmic (base 10) scale. D. Power spectrum at zero spatial frequency of the NFT simulation where spikes have been reconstructed, 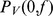. E. Spatial correlation function of the reconstructed voltage series from the NFT simulation. F. The spatio-temporal spectrum 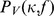 for the reconstructed voltage from the NFT simulation on a logarithmic (base 10) scale.

We now examine the power spectra for 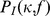 and 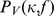. To complete comparisons, we have carried out simulations for the spike-based model, Eqs. (26)–(30), the NFT, Eqs. (31)–(34), and evaluated the theoretical field prediction through the transfer function 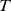 of Eq. (42). To consider the effect of the remnants of spike features on 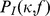, we also have constructed the power spectrum of a series of stereotypical spike features in 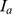, which can be added to the NFT predictions of 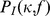. To illustrate these spectra, we have carried out simulations for the case of 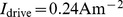, 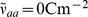, 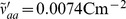 and 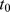 = 0.06 s. In Figs. 5 and 6 we show results for analyses of the current density term 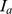 and membrane potential 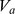, respectively.

In Fig. 5, the four rows, in order, represent analyses of the spike-based model, the simulations of the NFT equations, the theoretical analysis of the spectrum of the NFT through Eq. (42), and a theoretical analysis of NFT as for the third row but augmented with spike features. The three columns represent the breathing mode power 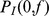, the spatial correlation function 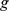 [from the inverse Fourier transform of 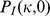] and the full spatio-temporal power spectrum 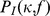. Panel A shows 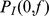 for the spike-based model. The power is large at zero frequency and falls with increasing frequency; however, it is dominated in this case by features related to the spike rate; namely peaks at about 22 Hz and its harmonics. Panel B shows the correlation function, showing that there is some significant spatial order in the system; with 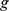 decaying to 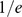 in about 2 cm. Panel C shows 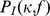; here we see that there are large features at 22 Hz and 44 Hz associated with spiking behavior superposed on a more smoothly varying background with a maximum at (0,0). The spatial frequency extent of these features, about 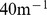, is equivalent to the correlation length seen in panel B. Panels D–F show the equivalent for the NFT simulation. The obvious difference is the lack of spike-features, since the NFT simulation does not contain spiking events. Otherwise, the shape (but not the magnitude) of the behavior is very similar to that of panels A–C. Panel G shows a theoretical calculation of 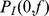 from Eq. (42); it is evident that it is very similar to that of the simulation of panel D. Panel H shows the spatial correlation function; it has a similar correlation length to those of B and E; however, it does not have the same minimum at approximately 0.07 m that is the case for panels B and E. This negative correlation in panels B and E may be attributable to the toroidal boundary conditions in space. Panel I shows 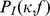, which agrees with the simulation of panel F and the background of the panel C for the spike-based model. Panel J depicts 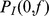 calculated from Eq. (42), with the addition of spike features arising from the spectrum of spikes. This compares well qualitatively to Panel A. The major discrepancy is the magnitude of the power. This is due to the interplay between the spike-based mode and the rate-based mode. The rate-based oscillation influences the synchrony of the spike-based mode, thus magnifying the power 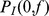 when resonances occur, such as for this set of parameters. The size of the major resonance can therefore vary tremendously as a function of 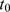. Panel K shows the NFT spatial correlation function, and panel L the NFT prediction of 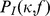 to which has been added the power spectrum due to a series of spike remnants in 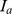. Panel L compares moderately well with panel C; the major discrepancy is the greater extent of the resonant features in 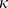, corresponding to less synchronization of neurons than is seen in the spike-based simulations in panel C. Overall, for Fig. 5, when features attributable to spikes are taken into account, we note that the NFT theory and simulation generally predict well the underlying shape of the power spectra (though not its magnitude).

In Fig. 6 we show a similar analysis for the membrane potential. The first row represents the simulation of the spike-based model; the second the reconstruction of a spike sequence from the simulations of the NFT model. Note that there is no NFT linearized prediction in this case since the NFT theory does not consider 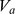 explicitly. The three columns represent the breathing mode power 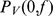, the spatial correlation function 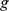 (from the inverse Fourier transform of 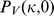) and the full spatio-temporal power spectrum 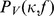. Panel A shows 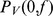 for the spike-based model. It is dominated by features related to the spike rate; namely peaks at about 22 Hz and its harmonics. Fluctuations due to non-spike (e.g. subthreshold) processes are much lower in magnitude. Panel B shows the spatial correlation function, showing, as in Fig. 5, 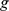 decaying to 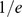 in about 2 cm. Panel C shows 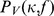; here we see that there are large features at 22 Hz and 44 Hz associated with spiking behavior; the spatial frequency extent of these (around 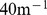) approximately equals the inverse of the spatial correlation length. Panels D–F show the equivalent for the NFT simulation, in which spikes have been generated through the process described earlier. One notes that Panel D shows a similar (but not exactly identical) spectrum to Panel A; for example, the spike rates are slightly different and the spikes are less broad, consistent with less variation in inter-spike interval. Panel E shows that there is no discernable correlation between neighboring neurons in this method. Panels F shows the spectrum 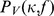 of the reconstructed spike sequence; the swaths at the spike rates represent very distinct firing frequencies that are uncorrelated in space. There is no equivalent NFT theoretical prediction since the NFT does not contain spiking events explicitly. Overall, for Fig. 6, we note that the NFT theory and simulation predict temporal structure of spiking well, but are not as accurate spatially. This is attributed to the problem of defining initial conditions from the reconstruction of spikes. One could in principle, knowing the result of the spike-based approach, define initial phases to produce a similar correlation. We have not done this. Spike rates also show more fluctuation in the spike-based model than the NFT model.

To summarize, we observe with Figs. 5 and 6 that the analysis of 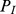 shown in Fig. 5 is more appropriate for analyzing the correspondence between the spike-based model and NFT where there is specific interest in the behavior of the NFT model (e.g. where collective modes dominate behavior). However, the latter analysis of 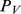 is likely to be appropriate when spike-rates are the dominant issue to consider.

We now consider how the behavior of the models change as key parameters are varied. To do this, we carry out simulations of both sets of equations (26)–(30) and (31)–(34) for the spike-based and NFT models, respectively; and use the methods of [14] to reconstruct spike-sequences from the NFT prediction. The power spectra 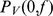 or 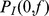 as appropriate for both situations are then compared. Plots of the power spectrum against temporal frequency are then stacked to represent graphically the changes in resonances and power fluctuations in response to a variation in a parameter.

### Dependence on External Current

**Figure 7:**
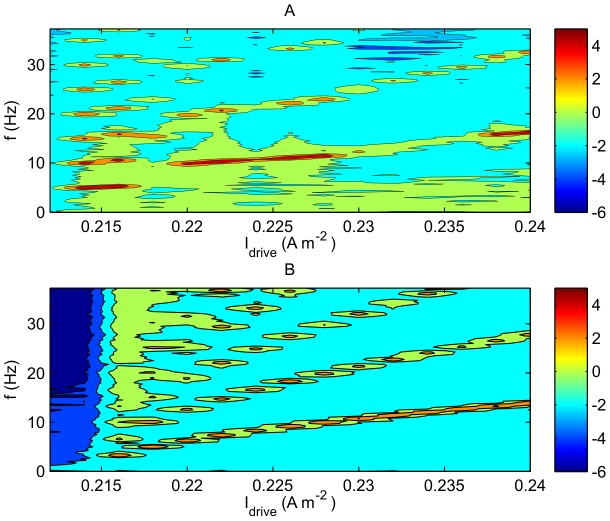
Comparison of spike-based and NFT models where spike rates are low. Power spectrum 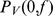 vs. drive current 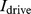 on a logarithmic (base 10) scale. Here 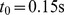, 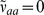, 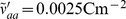. A. Spike based prediction. B. NFT prediction.

Fig. 7 demonstrates the effect of a change in the external driving current 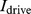. Higher 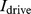 naturally leads to a higher firing rate. Part A shows the breathing mode power for the spike based model as a function of drive current. There is an abrupt change in the spectrum 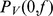 at 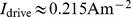; the maximum of 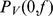 shifts from 5.5 Hz to 11 Hz. From this point, the major frequency feature increases in frequency as drive current increases, corresponding to a mean firing rate of the neurons that is in agreement with neural field theory. Part B shows the predictions of the neural field model in terms of the power spectrum 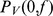 of the reconstructed voltage trace. The two graphs show that the resonances occur at similar frequencies, with a trend of increasing frequency with increasing 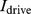. However, these frequencies are not exactly the same. For example, at 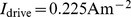, the spike-based approach in Panel A gives a simulated rate of 11 Hz, whereas in the NFT model shown in Panel B a rate of 9 Hz is observed. At 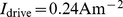, the spike-based and field-based frequencies are 17 Hz and 14 Hz respectively. In this case the spike-based model is dominated by the regular spiking behavior predicted by NFT. Note that the lumpy structure is a result of the resolution limit of the plot; it is not a chain of discrete peaks.

### Dependence on 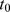 where Spiking Dominates

**Figure 8:**
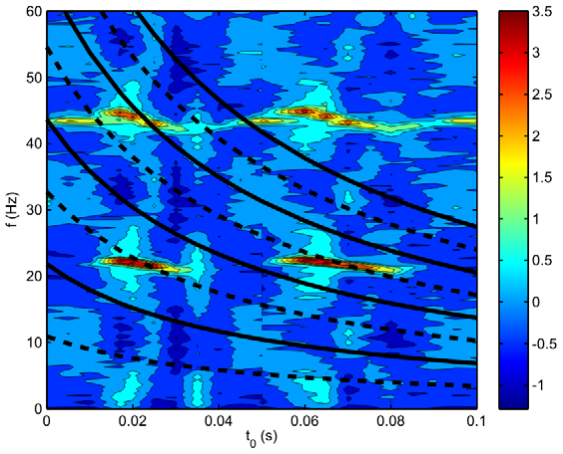
Breathing mode (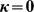) power vs. time delay 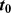 for a fast-firing case with the spike-based model. A plot of the power spectrum of 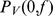 on a logarithmic (base 10) contour scale for the breathing mode (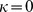) against time delay 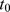 for a fast firing case. Here 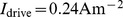, 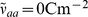, 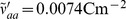. The solid lines show the resonances predicted by Eq. (43); the dashed lines the frequencies halfway between these.

In Fig. 8 we show the effect of varying the time delay 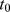 for the case of a high external drive current 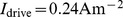 and fairly high loop feedback 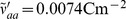. Since 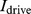 is well above 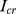 there is a high firing rate and the feedback is not required to maintain activity. Direct feedback is set to zero. The temporal frequency spectrum at 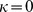 is plotted against time delay. Also shown on the plot are the predictions of resonances from the NFT, through Eq. (42). These are shown by the solid lines. The half-integer multiples of these are shown by the dashed lines. The plot clearly indicates a firing rate of around 22 Hz. A harmonic at around 44 Hz is also present on the plot. However, the firing rate is not completely independent of 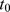, and varies between approximately 21 Hz and 22 Hz. There are two clear regions, at about 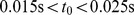 and 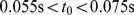 when the power is very large, indicating a sharp resonance in activity of the breathing mode, a property of the network.

A feature of this plot is the dependence on 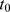 of the magnitude of the resonance at the spike-rate of 21–22 Hz. A large response occurs when the firing frequency is 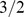 that of the fundamental predicted by Eq. (43). A low response occurs when the firing rate is exactly double that of the prediction of Eq. (43). We emphasize that in this case synaptic coupling between a neuron and its neighbors is weak compared with the driving current, implying that each neuron has a well established limit cycles for its firing, dominated by 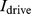. The reason for the discrepancy between the predicted resonances of Eq. (42) and the resonances seen in simulation appears to be the loss of spatial synchrony of the neurons at the time delays predicted by Eq. (42) to be resonances (i.e. the solid lines of Fig. 8). Instead of near synchronous firing, more intricate spatial patterns, e.g. traveling waves [47], [48], are formed causing a reduction in 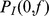. This phenomenon is not seen when spike rates are significantly reduced by lowering 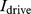, as discussed below.

### Dependence on 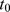 where Loop Effects Dominate

**Figure 9:**
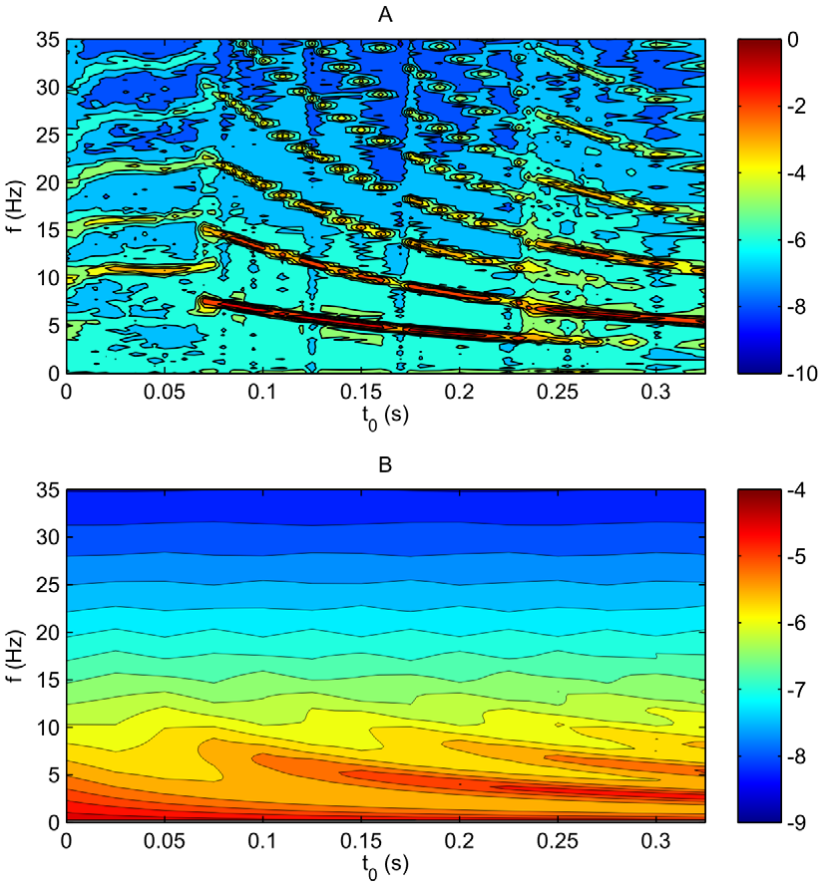
Power spectrum 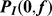 vs. time delay 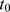 for a slow-firing case. Here, 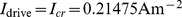, 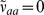, 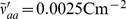. (A) Spike-based results. (B) Neural field results from linearized theory.

Next, the effect of a wide range of time delays is demonstrated for a low firing case. In order to elucidate the interaction between the loop resonances captured by the mean field approach and the effects of spike firings, a small loop connection strength 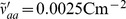 has been chosen, with no direct feedback, and a drive current equal to the critical current. This ensures that any positive feedback will result in significant activity. This time, the appropriate analysis is with the power in the current fluctuations, 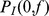. Part A of Fig. 9 shows the breathing mode power 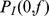 as a function of time delay 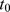, for a simulation of the spike based equations. Part B shows the same plot, but as predicted by the rate-based theory through Eq. (42).

One can see three clear regimes in Fig. 9A. For 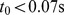, the major feature on the power spectrum is a resonance at about 10 Hz, which is *double* the neuron spike rate. However, there is a hint of power at 5 Hz at 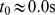. An examination of the neural firing patterns shows that there are strong correlations between neighboring neurons (e.g., a neuron firing at 5 Hz out of phase with its neighbor) leading to the 10 Hz feature being more prominent than the 5 Hz one. For 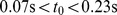, the major feature is the resonance induced by the delay loop. The neurons adopt a firing rate that is equivalent to the resonance frequency predicted by the NFT (i.e., the resonances of Fig. 9B). Harmonics of this frequency are also clearly visible. The key result in this graph is that the firing rates seen on Panel A for the spike-based model when 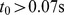 are in the positions predicted by NFT, while for 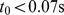 the NFT resonance is not strong enough to capture this mode and the firing rate reverts to that of the spike-based resonance.

**Figure 10:**
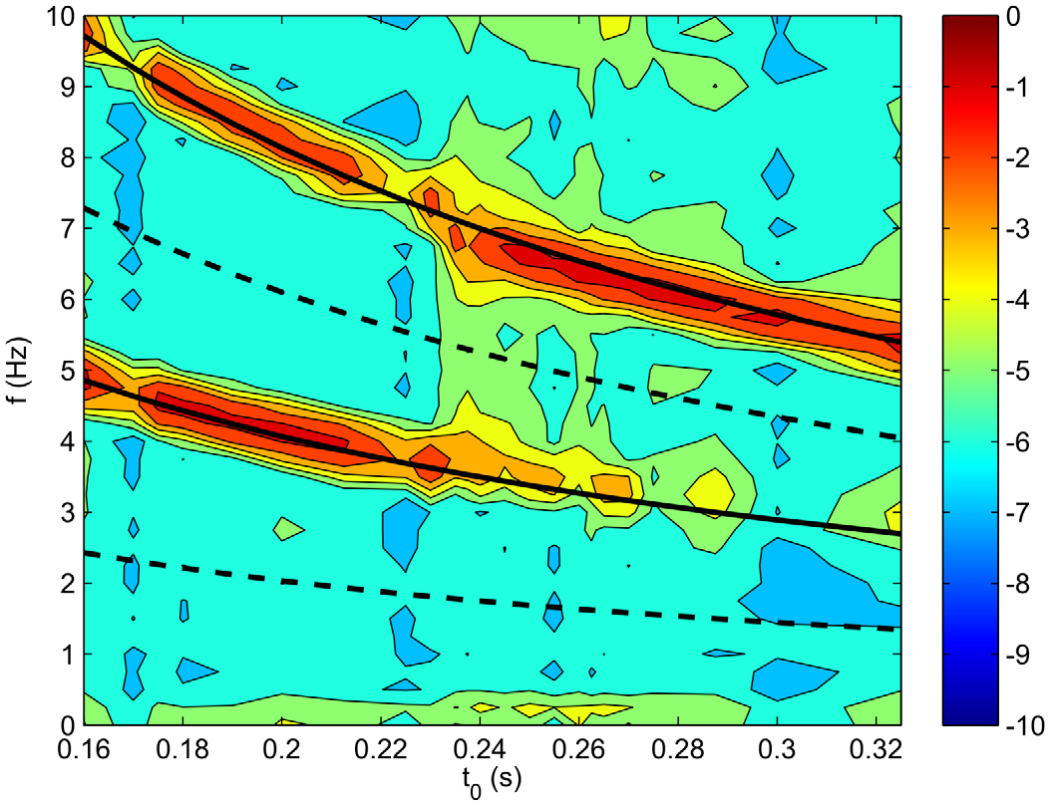
Enlarged view of the region of Fig. 9A for 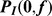 around 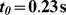. Solid lines represent resonances predicted by the neural field model through Eq. (43); dashed lines show the predicted minimums between resonances. The color denotes the logarithm (base 10) of the power.

A close-up of part of Fig. 9A is shown in Fig. 10, where the solid lines show the loop frequency prediction of Eq. (43); the two are very closely related. At 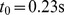 there is an abrupt change in the power spectrum 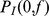 and for 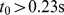 the lowest frequency peak falls in magnitude as 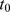 increases until it vanishes at 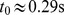. Here, the neurons are no longer able to fire slowly enough to follow the loop resonance frequency which is low for large 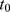, and instead the firing rate switches to (nearly) double the loop resonance frequency. However, this transition is subtle and Fig. 10 shows a very slight downward shift in the frequency compared to double the loop frequency.

The above behavior is similar to that found for a population-based neuron model with loop feedback [16]. In that model the authors found that their system could jump between two regimes of behavior as the time delay was varied. In one regime, the system fired with a rate equal to the reciprocal of the time delay, or an integer multiple of this frequency (i.e., it decreased as the time delay increased); in the other regime, it produced a firing rate independent of the time delay. The system alternated between these regimes as the delay time increased. In our model we also see this break between a firing rate roughly independent of time delay (for 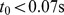), and one where the rate approximately follows the reciprocal of the delay time (for 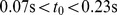).

### Dependence on Connection Strengths

The other major parameters that can be changed are the connection strengths. We illustrate this case by studying the effect of altering the balance between the direct and loop connection strengths 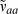 and 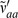, respectively.

The mean field solution for firing rate depends upon the sum of 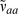 and 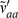. However, fluctuations in firing rate are expected to be different. By setting 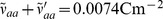, we ensure that the equilibrium firing rate is the same in all cases and we can study how the resonances and stability change as the balance between the terms changes. The other key parameters are selected as 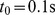 and 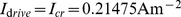.

**Figure 11:**
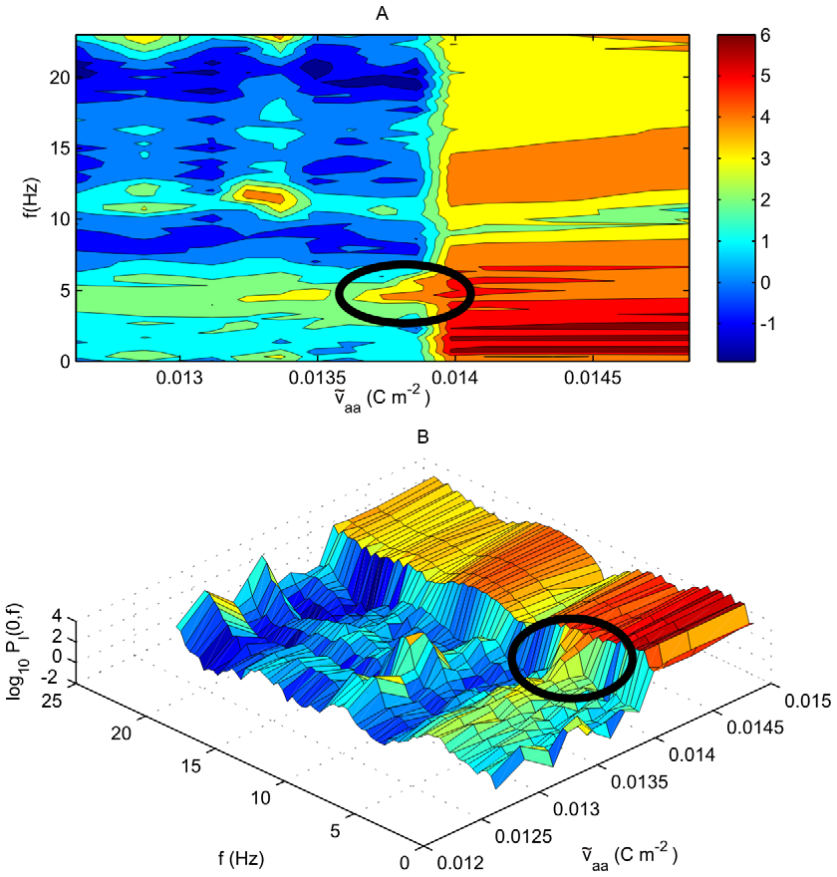
Power spectrum on a logarithmic (base 10) scale for the breathing mode (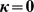) vs. direct connection strength 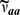 for a low-firing rate case. Here 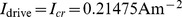, 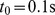, and 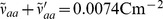. A. Power 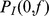 in the breathing mode. B. The same plot, shown in a three-dimensional form, with power shown on a base-10 logarithmic scale. The ellipses show the increase in power at 5 Hz before the bifurcation.

**Figure 12:**
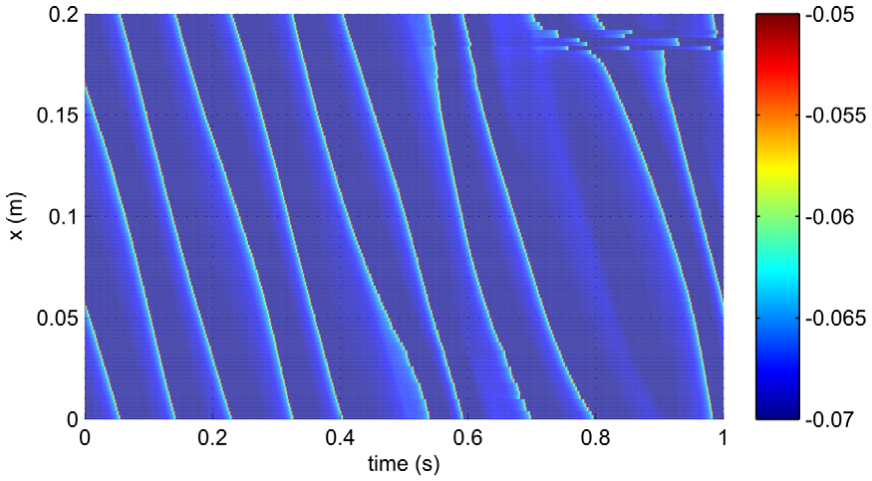
A section of the voltage 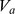 series in time and space for the case, 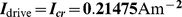, 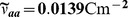, 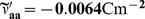, with 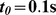.

Fig. 11 shows the breathing mode power 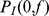 as a function of the direct (non-loop) connection strength 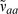. In Panel A power is plotted in the form of contours; Panel B shows a three-dimensional representation of the same information. Note that in the plot, 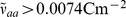, implying that the loop connection strength 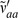 is negative. The most distinctive feature in this plot is a bifurcation at 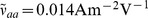, as a result of a strong loop negative feedback through 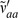. At 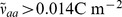, the system oscillates at about 5 Hz between a rapidly firing state and a non-firing state. For 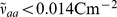, the system fires at about 12 Hz, as predicted by NFT. Close to bifurcation the system experiences large fluctuations in firing rate, as expected by NFT [49]. There is some evidence of an increase in power at about 5 Hz just before the bifurcation, for 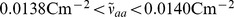. This part of the spectrum is indicated explicitly on both parts A and B of Fig. 11. The fluctuations for 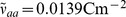 are shown explicitly in Fig. 12 which shows 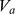 against time and space for one second of time. The plot shows propagating fronts of activity. The velocity of propagation has a range of approximately 0.4–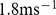 and a typical value of around 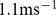, and this variation results in the firing rate of each neuron showing considerable fluctuation with time.

**Figure 13:**
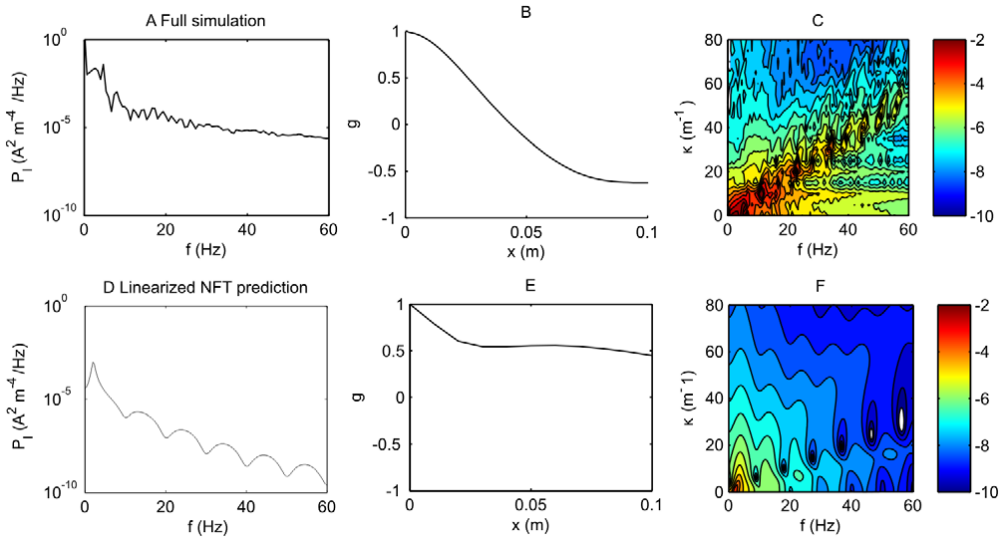
Comparison of the power spectra predicted from the spike based and neural field approaches close to the bifurcation. Here, 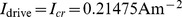, 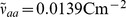, 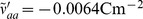, with 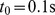. A. 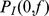 from the spike-based model B. The spatial correlation function of the spike-based model C. Results for 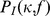 for the spike based model on a logarithmic (base 10) scale. D. Theoretical 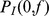 predicted by the transfer function Eq. (42) of the NFT model. E. Theoretical spatial correlation function predicted by the NFT model. F. Predictions of 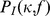 from the transfer function of the NFT model.

The point of bifurcation is also presented in more detail through Fig. 13. Panel A shows 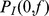; we see a decrease in power with increasing frequency, with a hint of a peak at around 4 Hz. Panel B shows the spatial correlation function 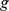; there is long-range order here with 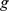 dropping to 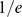 in about 3 cm. Panel C shows the 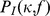 for the spike based simulation. There is a background of activity that peaks at (0,0); on top of this there is a diagonal line of peaks with gradient of approximately 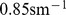; corresponding to a traveling wave with velocity of about 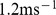, consistent with the typical velocity of a wavefront in Fig. 12.

This is significantly larger than the loop propagation speed 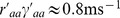 illustrating that the rate of propagation of activity along axons is not the sole determining factor for the wavefront velocity. Indeed, Bressloff has demonstrated that propagation of waves in a one-dimensional network of integrate-and-fire neurons is dispersive and dependent upon synaptic strength and delays in addition to axonal properties [50]. Panels D, E and F show the equivalent calculations from the NFT through Eq. (42). In panel D we see a power spectrum with a strong peak at 3 Hz (similar to the peak of panel A) and then clear resonances at higher frequencies. Panel E shows the correlation function 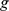; there is large long range order predicted, consistent with being on the edge of an instability. Panel F shows the predicted 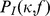. One can see a peak at about 3 Hz and zero spatial frequency, similar to Panel C for the spike-based simulation. However, the major feature of this plot are the deep dips lying on a line of gradient 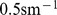. It is interesting to remark that this gradient is about half that seen for the resonances in Panel C. In these plots it is clear that the spike-based simulations and linearized NFT predictions are considerably different. This is not surprising given that this system lies very close to an instability where linear predictions are expected to break down. It is also possible that the critical point in NFT might be at a slightly different parameter value than for the spiking model.

## Discussion

We have explored the relationships between spiking-based and rate-based neural models by using both approaches to model the same test system — a one dimensional array of neurons coupled both directly and via a delayed feedback loop. The dynamics predicted by both approaches has been compared predominantly through the power spectra of the membrane potential 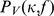 and current density 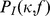. We have focused on the relationships between resonances associated with the firing of single cells and populations of cells, particularly the overlap and transitions between these two regimes. We have shown how the dynamics, especially prominent resonant effects, depend on the key parameters of the model (specifically delay-loop time, loop connection strengths and drive current density).

The spike-based approach of Eqs (26)–(30) supports two modes of oscillation. First, there is the highly nonlinear spiking mode in which each neuron spikes according to its input. This mode, for a single Wilson neuron, has been well-studied [2], [49], [51]. Also, a collective mode can exist, in which the firing rate undergoes small or large oscillations. The spectrum of these oscillations can be determined through neural field methods [as in Eq. (42)]. Both modes can be obtained through an analysis of an equivalent neural field model: the spiking modes from a reconstruction of the voltage, and demonstrated most directly through 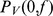; the collective modes through an analysis of the current fluctuations and demonstrated most directly using 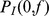.

At this point we again stress the distinction between the firing rate and fluctuations in the firing rate. Neural field theory predicts both, namely the firing rate 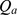 itself, and how the firing rate fluctuates with time and space. In an extreme case, this could take the form of bursting — a neuron fires rapidly for a short period of time, and then is silent for a period of time. Thus there are two *different* time scales here, the inverse of the firing rate, and the period for the bursting–silent oscillation. Generally, however, the collective modes are not of this extremely nonlinear bursting form, but can be modeled by the linear analysis of Eqs. (35)–(42).

Results of spike-based and NFT simulations and predictions can be compared through plots of the power spectrum in current density, 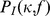 and membrane potential, 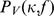. In the latter case, since the membrane potential does not feature in the NFT equations explicitly, a sequence must be reconstructed from knowledge of other variables, either the mean firing rate 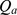 through time integration or the current density 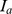 through a neuron-in-cell method [14]. Analysis of 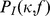, and particularly the breathing mode power 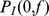 proves useful when there is significant power in the collective modes of oscillation; however, when spike-based behavior dominates, an analysis of 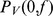 gives more direct insight. A disadvantage of analyzing the membrane potential is that the reconstruction of spike sequences from the NFT solution does not produce the spatial correlations that are predicted through 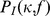 or seen in the spike-based model. This is because in the reconstruction of a voltage from a firing rate, [e.g., Eq. (46)] spatial effects manifest themselves differently; the reconstructed spike series depends upon the initial conditions which are not known a priori as a function of space.

The collective and spike-base modes are not entirely independent of each other, particularly when the two time scales are the same (or integer or half-integer multiples) of each other. Indeed, Wu et al. [16] found for a rate-based model of a Wilson neuron that receives feedback from itself, the behavior can be dominated by either type of resonance. At certain points a small change in parameters is sufficient to cause the behavior to switch between one resonance and the other. In this spatial model, we see similar behavior. However, the spatial dimension adds a complexity to the behavior that is not present in simpler models. This manifests itself for example in the intricate traveling-wave firing patterns that are demonstrated in Figs. 2 and 12 for example, that have been described by Osan et al. [47].

In some cases, the collective and spike-based modes are both present in a system, without significant signs of interaction. For example, Fig. 5 shows that the NFT predicts the underlying power spectrum, on which features due to spiking events sit. For example, in this case there is a peak at zero temporal and spatial frequency, as is frequent for neural field models away from a resonance condition (e.g., [39]). However, there are situations where one of the two modes can dominate In Fig. 7 the spiking mode dominates; neurons fire at a rate that is close to that predicted by the NFT but show little modulation in this rate — i.e. there is little collective oscillation present.

In Fig. 9A, and in more detail in Fig. 10, for 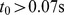, the collective oscillation dominates to the extent that it captures the spiking oscillation. This can be seen from the correspondence between the solid lines in Fig. 10 and the regions of high power. In this case the firing rate is no longer that predicted by the equilibrium value of 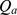 in Eq. (31), specifically 5.5 Hz, but is equal to the position of the predicted resonance in the rate. Specifically, the larger 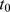, the lower the resonant frequency. We emphasize that this is not a trivial result. Resonances in spike rate 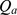 as predicted by NFT (i.e. the peaks in the spectrum 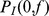) are not in general the same as the mean firing rate 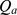 itself. Analysis of Fig. 9 requires the use of both the spike-based and rate-based paradigms in order to fully elucidate the results. Analysis of the spiking pattern (not shown) shows that the system is also highly synchronized in space — in other words the collective oscillation has entrained the spiking oscillation. This is consistent with the prior result that a particular firing-rate based model agreed with a corresponding integrate-and-fire spiking model when interactions were sufficiently slow [17]. In Fig. 9B, the predicted power for the NFT case shows clearly the NFT resonances moving to lower frequency as 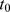 increases. It is also interesting to note the transition between different dominant modes of behavior in this system. For 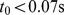, the strength of the collective oscillation is not sufficient to entrain the firing rate, which reverts to its equilibrium predicted value of 5.5 Hz. The major feature on Fig. 9A is at twice this, 11 Hz; analysis of the firing patterns (not shown) show that neurons are approximately paired; each fires in approximate anti-phase with its neighbor. Such behavior is common in neural simulations and its prevalence depends upon the strength of coupling between neurons, randomness in the couplings, noise and time delays. An anti-phase mode would be most likely when coupling strength and randomness are low [17], [52], noise is low [48], but for a small range of time delays [52], [53]. In our simulations we have not used random connections and have kept connection strengths low in order to ensure firing rates are of similar magnitude to resonances in the neural field simulations. Both of these favor the existence of an anti-phase state.

Fig. 9A is similar to the results seen by Wu et al. [16] in which a simpler spatially uniform model was shown to exhibit similar transitions between spike-based modes and collective modes of behavior as the loop delay was changed. In Ref. [16], spiking rates for one parameter set entrained alternately to either one of the two modes as 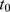 was increased. Several switches between the modes were observed as delay time was increased from 0 to 0.7 s. In the current study, delay times were limited to what is physical within a thalamocortical system, and only a single switch between a spike-based mode and a collective mode is observed. For a different parameter set, Wu et al. [16] also demonstrated a doubling of the primary frequency of oscillation with as a result of a small change in time delay, similar to the doubling observed in this study in Fig. 9A.

There can also be more complicated interplay between the two forms of oscillation. For example Fig. 8 demonstrates that the power in fluctuations in 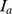 at a particular frequency can be strongly influenced by the relationship between this frequency (in this case 22 Hz) and the frequency of a collective resonance predicted by NFT shown in the figure by the solid and dashed lines. This variation in power requires particular comment. Naively, one might expect that where an NFT resonance, with positive gain, corresponds to the mean spike rate, there would be an enhancement of power. However, the opposite is the case here; the power 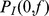 is much *reduced* when the resonance predicted by Eq. (43) corresponds to the spike rate. There are two points to discuss by way of explanation. First, resonances predicted by Eq. (43) are weak when 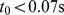, so interaction between the two might be expected to be small for this parameter range. Second, the spatial nature of the model is important. Consider the propagation of activity in space. When a neuron fires, there is a time frame 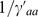 over which an effect is generated on the axon, through Eq. (30). There is then a delay time 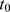 for the signal to traverse the thalamocortical loop, followed by a time 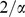 for an impact to be felt on the receiving neuron through Eq. (28). A signal therefore takes a time 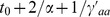 to return to the same neuron in the cortex; longer times allow for spatial propagation via the delay loop. At a spike rate of 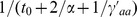 each neuron receives strong input that arose from itself one spike-interval in the past, and consequently spatial communication between neurons is relatively weak. The weak communication encourages the formation of a variety of spatially patterned states [17], [47], [52] which are not synchronized in space and therefore lead to a reduction in 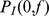. Such a pattern of alternating synchronous and asynchronous behavior has been found in previous studies [17].

It is possible that the dynamics of the spike based system is unduly influenced by the homogeneity of the parameters used [52]. For realistic systems, one would expect a wide range of values for the axonal lengths, synaptic decay times, etc. A system consisting of identical neurons may be particularly sensitive to modes of oscillation (e.g., synchronous in-phase or antiphase firing of all neurons) that are less likely to be seen in practice. Using homogeneous values in a model has the advantage of increased analytic tractability; however, implications of such homogeneity require further study. The methods discussed are easily generalizable to two dimensions with appropriate choice of wave equation (6). Results would be less easy to present, since two spatial dimensions and one temporal dimension would be present. Other neuron models (e.g. the bursting model used by Robinson et al. [15]) could be used by changing the forms of Eqs. (12)–(15), and finding the equivalent rate equation (16). An inhibitory population could be added with another set of variables.

To conclude, we remark that we have demonstrated considerable overlap between the spike-based and the neural-field approaches. Where neural-field resonances are strong, spiking rates can be entrained to these resonances. A system that allows both modes to feature can show interactions between the two. Both spike-based and rate-based paradigms must be used to fully analyze the system. A spatial dimension adds complexity to the situation discussed previously by Wu et al. [16]. The theories can be considered as complementary methods of approaching the neural modeling problem, each offering a different physical emphasis.
